# A Challenging Inflammatory Myopathy Case-Report: Dermatomyositis-Like Rash in an Anti-HMGCR Positive Patient with an Uncertain Muscle Histology

**DOI:** 10.31138/mjr.220823.imc

**Published:** 2023-08-22

**Authors:** Hugo Parente, Maria Pontes Ferreira, Catarina Soares, Emanuel Costa, Francisca Guimarães, Soraia Azevedo, Daniela Santos-Faria, José Tavares-Costa, Daniela Peixoto, Carmo Afonso, Filipa Teixeira

**Affiliations:** 1 Rheumatology Department, Unidade Local de Saúde do Alto Minho, Ponte de Lima, Portugal,; 2 Rheumatology Department, Hospital de Braga, Braga, Portugal

**Keywords:** Anti-HMGCR, heliotrope, necrotising myopathy, inflammatory myopathy

## INTRODUCTION

Idiopathic inflammatory myopathies (IIM) are an heterogenous group of rare immune-mediated diseases char-acterised by muscle weakness, elevation of muscular enzymes, auto-antibodies (aAbs) and inflammation on the biopsy tissue. They are divided in polymyositis (PM), dermatomyositis (DM), inclusion body myositis (IBM), anti-synthetase syndrome (ASS), overlap myositis (OM) and immune-mediated necrotising myopathy (IMNM).^1^ IMNMs are expressed with severe proximal muscle weakness (typically progressing faster and with more pain), high creatine kinase (CK) levels, predominant muscle fibre necrosis and no extramuscular features.^1^ The associated aAbs are anti-signal recognition (SRP) and anti-3-hydroxy-3-methylglutaryl-coenzyme A (HMGCR). The latter might be triggered by statin exposure. IMNMs’ diagnosis relies on its serological and histopathological aspects, and its treatment is harder to attain. We report herein a rare case of an admissible anti-HMGCR IMNM with DM-like cutaneous features, whose diagnosis was challenging due to its dubious histology.

## CASE REPORT

**Figure 1.**
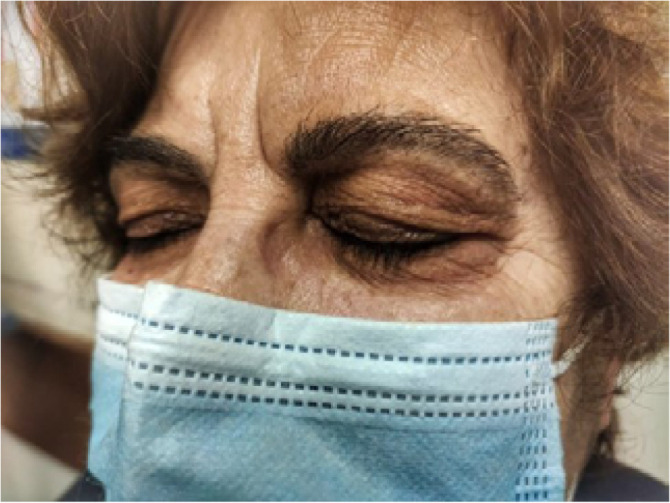
Bilateral heliotropic rash.

A 67-year-old patient was referred to the Rheumatology consultation for a 2-year proximal muscle weakness and pain, leading to limitation in her daily live activities - mainly brushing her hair and climbing stairs, having prompted some accidental falls. Her clinical background was positive for type 2 diabetes mellitus and dyslipidaemia for which she was treated with atorvastatin 10mg/day for 7 years prior. There was no family history of muscle or autoimmune diseases. The muscle symptoms were neither associated with a specific timing of her physical activities nor with carbohydrate/lipid ingestion. She had no constitutional, muco-cutaneous, oesophageal, or microvascular symptoms. Muscle strength examination revealed severe weakness. She had been treated 3 months earlier with intramuscular betamethasone 14mg, followed by a massive improvement in her symptoms. Her laboratory analysis revealed high CK levels (2212U/L), as well as myoglobin (1181U/L) and aldolase (22.3U/L) levels. Inflammatory markers were normal, so were urinalysis, antinuclear aAbs, and myositis-specific (anti-synthetase, anti-Mi2, anti-SAE, anti-MDA5) and associated aAbs (MAA - anti-RNP, anti-Ku, anti-PM-Scl, anti-SSa, anti-Ro52). The electromyography showed myopathic abnormalities, without neuropathic changes. Deltoid biopsy presented frequent muscle fibres at different stages of necrosis and two endomysial mixed CD3+ T lymphocyte inflammatory infiltrates, concluded to be common features of both PM and IMNM. Cancer screening done by thoraco-abdominopelvic computed tomography, mammography, positron emission tomography scan, esophagogastroduodenoscopy and colonoscopy was negative. She was started on prednisolone 1mg/kg/day and, by the end of the first month, her symptoms and lab results had improved (CK 728U/L, myoglobin 646U/L). At that timing, the patient was found to have a bilateral heliotropic rash (**Figure 1**). The aAbs against TIF1y and NXP2 were negative. At the second month of corticosteroids, while tapering, her symptoms aggravated, so she started azathioprine 50mg/day and stopped taking atorvastatin. Later, the results for the first-time requested aAbs related to IMNM came negative for SRP and positive for HMGCR. As adverse events for corticosteroids started to compound, namely cataracts, hyperglycaemia, and osteoporosis, she was proposed treatment with intravenous immunoglobulin (IVIg) 2g/kg for 5 days, followed by monthly booster doses of 1g/kg spread over 3 days, while maintaining corticosteroids and azathioprine (started, respectively, 4 and 3 months previously). After only 1 month of treatment, she noted greater muscle strength and better overall functionality, with resolution of the heliotropic rash. The muscle enzymes were normal. At the last follow-up, after almost 9 months, and with an ongoing decreasing corticosteroid dose of 10mg/day, there was no evidence of disease recurrence.

## DISCUSSION

IMNMs can be subdivided into anti-SRP, anti-HMGCR and seronegative. These last two might be more frequently associated with malignancies. Anti-HMGCR aAb was first identified in 2010 among IMNM patients, aimed at HMGCR antigen, a key enzyme in cholesterol synthesis that is targeted by statins.^3^ These aAbs’ pathogenicity is accepted, as in vitro they induce muscle atrophy impairing muscle regeneration.^2^ Also, their titres correlate with disease activity.^4^ They are found to be highly specific for immune-mediated myopathies, since they are not found in the majority of statin-exposed individuals, including those with a self-limited myopathy.^5^ Anti-HMGCR primarily associates with IMNM and rarely with other idiopathic inflammatory myopathies (IIM) and connective tissue diseases.^6,7^ Anti-HMGCR IMNM typically affects people between 40 and 60 years old, with a female preponderance.^3^ Statin exposure covers about 50 to 66% of these patients, and their CK levels remain elevated for a mean duration of 39 months.^8^ This elevation has been shown to actually precede muscle weakness by months to years in an atorvastatin-associated anti-HMGCR IMNM cohort,^8^ so that one should prompt HMGCR aAb search if levels are persistent despite statin suspension. Extramuscular manifestations are usually absent.^2–4^ As for the anatomo-pathological aspects, it reiterates necrosis, muscle atrophy and variable grades of regeneration, with little to no inflammatory infiltrates.^2,4^ There are only four^9–12^ reported cases of statin-induced anti-HMGCR IMNM with typical cutaneous DM rash. Although our patient displays a histology with mixed IIM characteristics, those are not of DM, and it has no other clinical or serological arguments that fully support the OM diagnosis. Overlap myositis includes IIM plus symptoms peculiar of systemic lupus erythematous, systemic sclerosis or Sjögren’s disease, and is usually linked with MAAs like anti-Ku, anti-PM/Scl, anti-Ro52 and anti-U1-RNP.^13^ This rationale strengthens our confidence in this case’s diagnostic assumption of an anti-HMGCR IMNM with DM cutaneous involvement. The previously mentioned cases reported patients between 47 and 61 years old exposed to statins 8 months to 10 years prior to their symptoms. Proximal muscle weakness and DM rashes were observed for 4 weeks to 4 years. None of the muscle biopsies showed any sign of dermatomyositis. Two of these patients were submitted to skin biopsy, showing neutrophilic interface dermatitis. All patients improved significantly within 2–12 months of high-dose corticosteroids, immunosuppressive agents and IVIg. Statin discontinuation and corticosteroid therapy by themselves are insufficient for anti-HMGCR IMNM,^8^ and the 2016 European Neuromuscular Centre (ENMC) International Workshop has proposed analogous treatment recommendations for anti-HMGCR IMNM.^14^ The triple therapy composed of corticosteroids, immunosuppressants (methotrexate, azathioprine, cyclophosphamide, or mycophenolate mofetil) and IVIg has been suggested.^15,16^ Several studies advocated IVIg at a dose of 2g/kg spread over 2-5 days,^17,18,19^ which can then be stopped or tapered as tolerated.^14^ According to the Criteria for Clinical Use of Immunoglobulin in Australia, regarding inflammatory myopathies, following the induction dose of 2g/kg in 2 to 5 divided doses, maintenance should be achieved with a maximum dose of 1g/kg monthly. We have kept a monthly low-dose protocol that will be tapered as soon as corticosteroids are at a stable low-dose. The idiosyncrasy of our case lies also on its chronicity, which is not usually observed for IMNM. We could not comply, however, with a classic DM diagnosis, since there is no histological evidence for it (as sarcoplasmic MxA expression),^20^ and the anti-HMGCR frequency is remarkably superior for IMNM. Our diagnosis was, thus, based on the muscle biopsy (albeit the blended inflammation), statin exposure and anti-HMGCR aAb positivity.

This validated association between anti-HMGCR IMNM and DM cutaneous traits implies that patients with this kind of rashes should be cautiously evaluated, even more so as anti-HMGCR aAbs are not routinely included in most myositis panels. Cutaneous DM-like highlights might just be the lead to anti-HMGCR IMNM, especially in patients with severe proximal myalgias and weakness, and with statin exposure. Clinical, serological, and histological components have to be integrated altogether.

## References

[1] Benveniste O Stenzel W Allenbach Y. Advances in serological diagnostics of inflammatory myopathies. Curr Opin Neurol 2016;29(5):662–73. 27538058 10.1097/WCO.0000000000000376

[2] Allenbach Y Benveniste O. Peculiar clinicopathological features of immune-mediated necrotizing myopathies. Curr Opin Rheumatol 2018;30(6): 655–63. 30239349 10.1097/BOR.0000000000000547

[3] Mammen AL Chung T Christopher-Stine L Rosen P Rosen A Doering KR Autoantibodies against 3-hydroxy-3-methylglutaryl-coenzyme A reductase in patients with statin-associated autoimmune myopathy. Arthritis Rheum 2011;63(3):713–21. 21360500 10.1002/art.30156 PMC3335400

[4] Allenbach Y Drouot L Rigolet A Charuel JL Jouen F Romero NB Anti-HMGCR autoantibodies in European patients with autoimmune necrotizing myopathies: inconstant exposure to statin. Medicine (Baltimore) 2014;93(3):150–7. 24797170 10.1097/MD.0000000000000028 PMC4632910

[5] Mammen AL Pak K Williams EK Brisson D Coresh J Selvin J Rarity of anti-3-hydroxy-3-methylglutaryl-coenzyme A reductase antibodies in statin users, including those with self-limited musculoskeletal side effects. Arthritis Care Res (Hoboken) 2012;64(2):269–72. 21972203 10.1002/acr.20662 PMC3415973

[6] Musset L Allenbach Y Benveniste O Boyer O Bossuyt X Bentow C Anti-HMGCR antibodies as a biomarker for immune-mediated necrotizing myopathies: a history of statins and experience from a large international multi-center study. Autoimmun Rev 2016;15(10):983–93. 27491568 10.1016/j.autrev.2016.07.023

[7] Hudson M Luck Y Stephenson M Choi MY Wang M Baron M Anti-HMGCR antibodies in systemic sclerosis. Medicine (Baltimore) 2016;95(44): e5280. 27858897 10.1097/MD.0000000000005280 PMC5591145

[8] Troyanov Y Landon-Cardinal O Fritzler MJ Ferreira J Targoff IN Rich E Atorvastatin-induced necrotizing autoimmune myositis: an emerging dominant entity in patients with autoimmune myositis presenting with a pure polymyositis phenotype. Medicine (Baltimore) 2017;96(3):e5694. 28099331 10.1097/MD.0000000000005694 PMC5279076

[9] Lavian M Mozaffar T Goyal N. Clinical dermatomyositis associated with anti-HMG-CoA reductase antibody positive immune mediated necrotizing myopathy: a case report (P2.125). Neurology 2017;88(16 Supplement):P2125.

[10] Merlant M Fite C Kottler D Maisonobe L Dossier A Deschamps L [Dermatomyositis-like syndrome revealing statin-induced necrotizing autoimmune myopathy with anti-HMGCR antibodies]. Ann Dermatol Venereol 2019;146(8–9):550–6. 30929872 10.1016/j.annder.2018.12.010

[11] Parikh P Tavee J Soltanzadeh P Mammen AL McKeever P Li Y. Anti-3-hydroxy-3-methylglutaryl-coenzyme a reductase autoantibody-positive necrotizing autoimmune myopathy with dermatomyositis-like eruption. Muscle Nerve 2018;57(6):E135–E136. 29346706 10.1002/mus.26072

[12] Lim D Landon-Cardinal O Ellezam B Belisle A Genois A Sirois J Statin-associated anti-HMGCR immune-mediated necrotizing myopathy with dermatomyositis-like features: A case report. SAGE Open Med Case Rep 2020 Dec 29;8:2050313X20984120. doi: 10.1177/2050313X20984120. PMID: ; PMCID: . 33447390 PMC7780312

[13] Benveniste O Stenzel W Allenbach Y. Advances in serological diagnostics of inflammatory myopathies. Curr Opin Neurol 2016;29:662–73. 27538058 10.1097/WCO.0000000000000376

[14] Allenbach Y Mammen AL Benveniste O Stenzel W. 224th ENMC international workshop: clinico-sero-pathological classification of immune-mediated necrotizing myopathies Zandvoort, The Netherlands, 14–16 October 2016. Neuromuscul Disord 2018;28(1):87–99. 29221629 10.1016/j.nmd.2017.09.016

[15] Mammen AL. Statin-Associated Autoimmune Myopathy. N Engl J Med 2016;374:664–9. 26886523 10.1056/NEJMra1515161

[16] Basharat P Lahouti AH Paik JJ Albayda J Pinal-Fernandez I Bichile T Statin-Induced Anti-HMGCR-Associated Myopathy. J Am Coll Cardiol 2016;68:234–5. 27386780 10.1016/j.jacc.2016.04.037 PMC5640444

[17] Mulhearn B Bruce IN. Indications for IVIG in rheumatic diseases. Rheumatology 2015;54(3):383–91. 25406359 10.1093/rheumatology/keu429 PMC4334686

[18] Wang DX Shu XM Tian XL Chen F Zu N Ma L Intravenous immunoglobulin therapy in adult patients with polymyositis/dermatomyositis: A systematic literature review. Clin Rheumatol 2012;31(5):801–6. 22274797 10.1007/s10067-012-1940-5

[19] Kampylafka EI Kosmidis ML Panagiotakos DB Dalakas M Moutsopoulos HM Tzioufas AG. The effect of intravenous immunoglobulin (IVIG) treatment on patients with dermatomyositis: A 4-year follow-up study. Clin Exp Rheumatol 2012;30(3):397–401. 22510247

[20] Uruha A Allenbach Y Charuel JL Musset L Aussy A Boyer O Diagnostic potential of sarcoplasmic myxovirus resistance protein A expression in subsets of dermatomyositis. Neuropathol Appl Neurobiol 2019;45(5):513–22. 30267437 10.1111/nan.12519

